# Hollow Mesoporous Carbon Spheres for High Performance Symmetrical and Aqueous Zinc-Ion Hybrid Supercapacitor

**DOI:** 10.3389/fchem.2020.00663

**Published:** 2020-09-15

**Authors:** Sihan Chen, Gaoqi Yang, Xiaojuan Zhao, Nengze Wang, Tingting Luo, Xu Chen, Tianci Wu, Shijie Jiang, Peter A. van Aken, Shile Qu, Tao Li, Liang Du, Jun Zhang, Hanbin Wang, Hao Wang

**Affiliations:** ^1^Hubei Key Laboratory of Ferro and Piezoelectric Materials and Devices, Faculty of Physics and Electronic Science, Hubei University, Wuhan, China; ^2^State Key Laboratory of Advanced Technology for Materials Synthesis and Processing, Wuhan University of Technology, Wuhan, China; ^3^Stuttgart Center for Electron Microscopy, Max Planck Institute for Solid State Research, Stuttgart, Germany

**Keywords:** mesoporous carbon, zinc ion battery, supercapacitor, hollow sphere, energy storage

## Abstract

Zinc–ion hybrid supercapacitors are a promising energy storage device as they simultaneously combine the high capacity of batteries and the high power of supercapacitors. However, the practical application of Zinc–ion hybrid supercapacitors is hindered by insufficient energy density and poor rate performance. In this study, a symmetrical zinc–ion hybrid supercapacitor device was constructed with hollow mesoporous-carbon nanospheres as electrode materials, and aqueous ZnSO_4_ adopted as an electrolyte. Benefiting from the mesoporous structure and high specific area (800 m^2^/g) of the hollow carbon nanospheres, fast capacitor-type ion adsorption/de-adsorption on both the cathode and the anode can be achieved, as well as additional battery-type Zn/Zn^2+^ electroplating/stripping on the anode. This device thus demonstrates outstanding electrochemical performance, with high capacity (212.1 F/g at 0.2 A/g), a high energy density (75.4 Wh/kg at 0.16 kW/kg), a good rate performance (34.2 Wh/kg energy density maintained at a high power density of 16.0 kW/kg) and excellent cycling stability with 99.4% capacitance retention after 2,500 cycles at 2 A/g. The engineering of this new configuration provides an extremely safe, high-rate, and durable energy-storage device.

## Introduction

With the continuous development of electric vehicles, smart electric grids, and miniaturized electronics, it is important to develop high–performance electrochemical energy storage systems (Sun et al., 2018; Zhang et al., 2019). The construction of better energy storage devices relies not only on the structure design of electrode materials, but also more crucially, it depends on the engineering of the device configuration (Zhang et al., 2014; Zuo et al., 2017; Chen et al., 2019b).

A hybrid battery-supercapacitor device, which is typically constructed with a high capacity battery–type electrode and a high rate capacitor–type electrode, has proven to be an effective way of simultaneously combining the merits of batteries and supercapacitors (Gan et al., 2019; Wang et al., 2019; Tan et al., 2020). Currently, existing electrolytes include organic, ionic liquid, and aqueous solutions, among which the later has advantages of high ionic conductivity, low cost, inflammability, and it is environmentally benign (Chen et al., 2014; Wan et al., 2018). Energy storage systems based on monovalent cations (Li^+^, Na^+^, and K^+^) have been widely investigated and significant progress has been achieved (Shen and Yu, 2017; Shen et al., 2018, 2019). Multivalent cations (Mg^2+^, Zn^2+^, Ni^2+^, Ca^2+^, and Al^3+^) are becoming more attractive, since one mole reacted multivalent ions can provide double or triple the number of electrons as compared to monovalent cations (Bitenc and Dominko, 2018; Cui et al., 2018; Dong et al., 2018; Liu et al., 2019a,b; Zhan et al., 2020). Moreover, multivalent cation-based devices are also more air–resistant and thus more applicable in practice (Sun et al., 2020).

Aqueous zinc–ion hybrid supercapacitors (ZHSs) have recently emerged as promising energy storage devices, due to the intrinsic advantages of the zinc element, such as a high capacity of 820 mAh/g in theory, low redox potential of −0.76 V vs. a standard hydrogen electrode (Ma et al., 2018; Chen et al., 2019a; Yu et al., 2019). For instance, Ma et al. (2019) have developed a ZHS with a γ-phase MnO_2_ nanorods as a cathode and activated carbon particles as an anode, which demonstrated a high specific capacity of 54.1 mAh/g (34.8 Wh/kg) at 0.1 A/g current density. After 1,000 charge/discharge cycles, 80% of the device capacity is preserved. Dong et al. have constructed a novel ZHS device using Zn metal and activated carbon as an anode and a cathode, respectively. This device revealed a high capacity of 121 mAh/g (84 Wh/kg) at 0.1 A/g current density, meanwhile, a high cycling stability of 91% capacity retention after 10 k charge/discharge cycles at 1 A/g (Dong et al., 2018). At the same time, Wang et al. made a similar ZHS device using Zn-metal as anode and activated-carbon as a cathode with a capacity of 170 F/g at 0.1 A/g, an energy density of 52.7 Wh/kg at 1.7 kW/kg and a 91% capacitance retention after 20 k cycles at 2 A/g (Wang et al., 2018).

In this study, a symmetrical zinc–ion hybrid supercapacitor system was constructed using mesoporous-carbon nanospheres with a hollow structure as both anode and cathode materials, as well as aqueous ZnSO_4_, which was adopted as an electrolyte. The device chemistry is based on the adsorption or de-adsorption of anions onto mesoporous carbon as the cathode reaction, and electroplating or stripping of Zn/Zn^2+^ as the anode reaction. The capacity of the symmetry zinc–ion hybrid supercapacitor device reaches 212.1 F/g at a current density of 0.2 A/g, corresponding to an energy density of 75.4 Wh/kg at a power density of 0.16 kW/kg. When increasing the current density to 20 A/g, 34.2 Wh/kg energy density is maintained at a high power density of 16.0 kW/kg, which yields a good rate capability. Te device exhibits excellent cycling stability with 99.4% capacity retention over 2,500 cycles.

## Experimental Sections

### Synthesis of Resorcinol–Formaldehyde (RF) Coated SiO_2_ (SiO_2_@RF) Spheres

SiO_2_@RF spheres were one–step synthesized by a modified liquid deposition method (Fang et al., 2014). In a typical process, 3.46 mL of tetrapropoxysilane (TPOS) was dissolved into a mixture solution of ethanol (70 mL), deionized water (10 mL), and ammonia solution (3 mL). Then 0.4 g of resorcinol and 0.56 mL of formaldehyde were sequentially added into the solution, and then magnetically stirred at 35°C for 24 h. SiO_2_@RF spheres were separated from the obtained suspension by suction filtration, washed with water/ethanol, and dried at atmosphere.

### Synthesis of Hollow Mesoporous-Carbon Spheres (HMCS)

The SiO_2_@C nanospheres were obtained by carbonization of SiO_2_@RF at 700°C for 5 h under a N_2_ atmosphere, with a heating rate of 2°C/min. After washing with 4 M NaOH solution, the as-synthesized core-shell SiO_2_@C spheres were converted into the HMCS.

### Materials Characterization

X-ray powder diffraction (XRD) was performed with a Bruker D8 diffractometer with Cu Kα radiation. The size and morphology of as-synthesized materials were characterized by Scanning Electron Microscope (SEM, JSM7100F JEOL). Transmission electron microscopy (TEM), high-resolution TEM (HRTEM), and high-angle annular dark-field (HAADF) scanning transmission electron microscopy (STEM) analysis were performed using a JEOL ARM 200F microscope equipped with a cold field emission electron source and an image Cs corrector (CEOS GmbH) operated at 200 kV. The specific surface area of HMCS was measured using a Micromeritics ASAP2020 and calculated using the Brunauer-Emmett-Teller (BET) method. X-ray photoelectron spectroscopy (XPS) measurements were carried out using a Thermo Fisher Scientific Escalab 250Xi spectrometer with Al Kα radiation.

### Electrochemical Measurements

Electrochemical measurements were performed *via* an electrochemical workstation of CHI760E. To prepare the electrode materials, carbon nanospheres, and acetylene black were firstly uniformly dispersed in a poly-tetra–fluoroethylene (PTFE) suspension (60 wt%) into a slurry under ultrasonication for 1 h. The ratio of electroactive materials, binder, and conducting materials was controlled to be 8:1:1. Then, the slurry was coated on a nickel foam disc with a diameter of 12 mm, followed by drying at 70°C under vacuum for 12 h. After that, the nickel foam with electroactive materials was pressed into a plate under 10 MPa. The loading of the electroactive materials was controlled to be 3–4 mg/cm^2^ for each electrode. Symmetric hybrid supercapacitor devices were assembled with a standard coin battery set-up, with the as-prepared electrodes described above, as both anode and cathode materials. Three different electrolytes were adopted to study the energy-storage mechanism and to optimize the electrochemical performance, including 2 M ZnSO_4_, 1 M Na_2_SO_4_, and their combination. Cyclic voltammetry (CV) and galvanostatic charge/discharge (GCD) measurements were applied to characterize the electrochemical performance of the constructed devices.

## Results and Discussions

**Figure 1:**
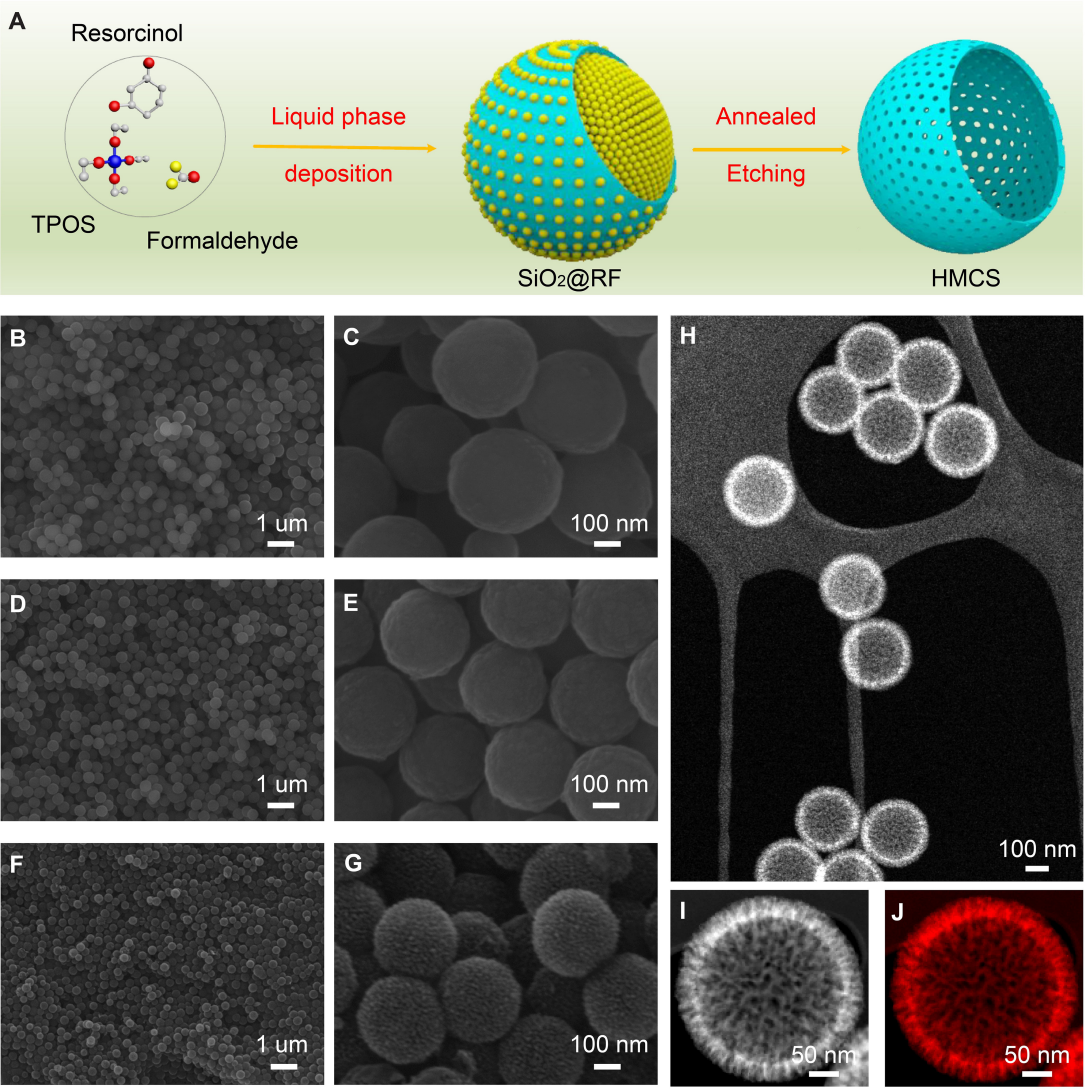
**(A)** Illustration of the fabrication processes of hollow mesoporous carbon spheres. Low– and high–magnification SEM images **(B,C)** of resorcinol-formaldehyde-coated SiO_2_ nanospheres intermediates, **(D,E)** carbon-coated SiO_2_ intermediates after carbonizing the organic surface, and **(F,G)** carbon nanospheres, respectively. **(H)** HAADF image of as-synthesized carbon nanospheres, which shows obvious hollow spherical structures. **(I)** A magnified STEM image of one single carbon sphere with a porous surface and **(J)** C-*K* edge elemental mapping.

The preparation process of HMCS is illustrated in Figure 1A. Firstly, core-shell SiO_2_@RF nanospheres are synthesized as an intermediate by a liquid deposition method containing TPOS, formaldehyde, and resorcinol. Then, SiO_2_@RF is carbonized into SiO_2_@C at 700°C under N_2_ for 5 h, and HMCS is obtained after removing SiO_2_ by NaOH etching. The SEM images of SiO_2_@RF demonstrate (Figures 1B,C) that the product has a spherical shape with a high uniformity of monodisperse spheres, with an average diameter of about 350 nm. A layer of mesoporous silica formed on the surface of SiO_2_@RF. Figures 1D,E are SEM images of SiO_2_@C nanospheres, revealing that the morphology of SiO_2_@RF is well-maintained after annealing at 700°C, with the carbonization process under precise control. Figures 1F,G show a uniform and spherical shape of as-synthesized HMCS, but their size decreases to about ~310 nm in diameter. The surface roughness increases after alkaline etching of SiO_2_@C. From SEM analysis, HMCS has a relatively rough surface, which can provide more active sites to modify functional groups on their surface, effectively avoiding the phenomenon of pore blockage. This structure is therefore very advantageous for the rapid adsorption/desorption of electrolyte ions.

STEM techniques were used to characterize the inner structure and elemental distribution of HMCS particles. A low–magnification STEM–HAADF image (Figure 1H) further proves that the HMCS particles are uniform, non-agglomerated and possess an obvious hollow structure with an inner diameter of ~235 mm and an outer diameter of ~310 nm. Interpenetrating channels of the HMCS surface are observed in a magnified STEM–HAADF image (Figure 1I). The element carbon is uniformly distributed throughout the sphere as presented in the STEM–EELS image (Figure 1J). The surface composition of the hollow mesoporous carbon spheres was analyzed by XPS (Figure S1). The C-1s spectrum has been deconvoluted into three peaks at 284.6, 286.1, and 288.3 eV, which are assigned to C–C, C–O, and C=O, respectively, based on XPS spectrum analysis. These results confirm the complete carbonization of SiO_2_@RF, and SiO_2_ removal by NaOH afterward. The hollow and porous surface structures of carbon nanospheres provide ultrahigh porosity and excellent performance of molecular sieving, which makes it possible for them to interact with atoms, molecules, and even larger objects, not only on their outer surface but also throughout the entire material, which can benefit its electrochemical performance as an electroactive material.

**Figure 2:**
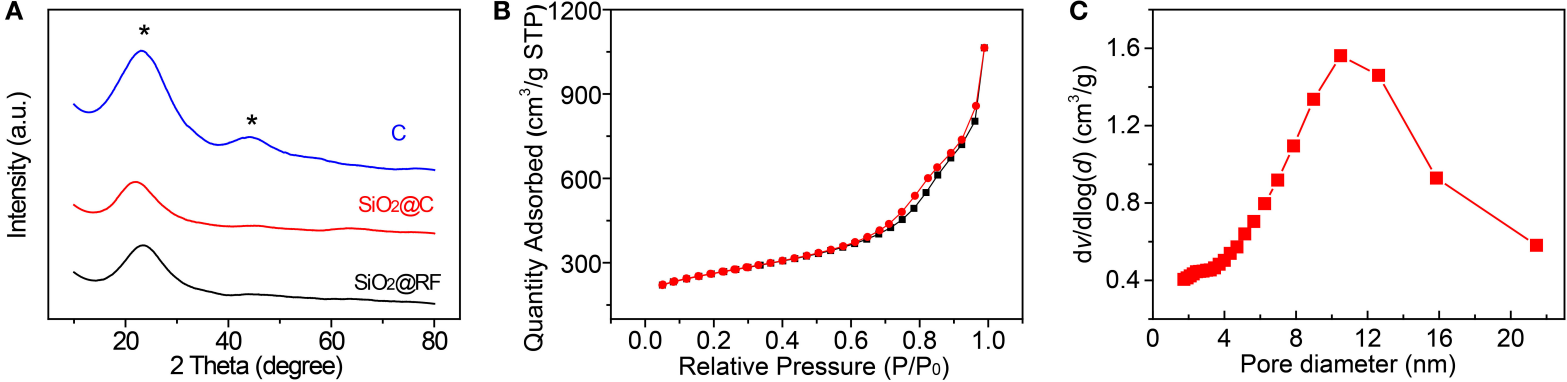
**(A)** The XRD spectra of SiO_2_@RF, SiO_2_@C, and carbon nanospheres. **(B)** N_2_ adsorption-desorption isotherms and **(C)** pore size distribution of carbon nanospheres. The star means diffraction peaks of Carbon.

Figure 2A shows X-ray diffraction patterns of the reaction intermediates during synthesis and the HMCS spheres. The XRD patterns of SiO_2_@RF, SiO_2_@C, and HMCS show all an obvious diffraction peak at around 24°. The 24° diffraction peak (2θ) corresponds to the (002) crystal plane of graphite. The characteristic diffraction peak intensity of HMCS increases and the peak is sharper, indicating that the crystallinity has increased, and the conductivity has improved after high-temperature calcination. In addition, a broad diffraction peak appeared near 43° in the diffraction pattern of HMCS, which corresponds to the (101) crystal plane of graphite, illustrating that the as-prepared carbon material has crystalline graphite characteristics. No further diffraction maxima apart from the two mentioned before were observed.

Figure 2B shows the N_2_ adsorption-desorption isotherm of the as-synthesized HMCS. A typical Type-IV isotherm can be seen with an H3-type hysteresis loop at the relative pressure of P/P_0_ > 0.7, according to the IUPAC classification, which indicates the mesoporous-rich textural and slit-pore properties of the carbon nanospheres. Pore size distribution is then extracted from the isotherm curve, as shown in Figure 2C, which shows a mean pore diameter of about 10 nm. The specific area of carbon spheres can also be calculated with a value of 800 m^2^/g, which is much higher than the 427 m^2^/g of mesoporous carbon spheres reported by the Zhao group (Yan et al., 2007). This mesoporous structure and slit pore surface of the carbon spheres are beneficial for ion diffusion in the electrolyte, which improves the electrochemical performance of the electrode materials.

**Figure 3:**
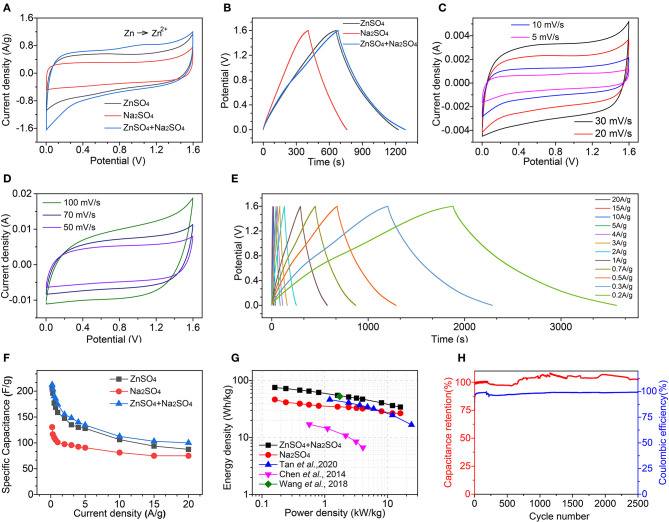
Electrochemical performance of symmetric electrochemical energy storage devices, constructed by as-synthesized HMCS as electroactive materials and three different electrolytes, 1 M Na_2_SO_4_, 2 M ZnSO_4_, and their mixture. **(A)** CV curves of these three devices at 5 mV/s. **(B)** Charge–discharge curves at 0.5 A/g. **(C,D)** CV curves and **(E)** charge-discharge curves at different scan rates or current densities, respectively. **(F)** The calculated specific capacities at different current densities. **(G)** Ragone plots of the electrochemical energy-storage devices and compared to previously reported results (Chen et al., 2014; Wang et al., 2018; Tan et al., 2020). **(H)** The cyclic stability and columbic efficiency of the devices.

To construct efficient zinc–ion hybrid supercapacitors, as-synthesized HMCS particles are adopted as electroactive materials for both the anode and cathode, with three different electrolytes of 1 M Na_2_SO_4_, 2 M ZnSO_4_, and their mixture. Figure 3A shows the CV curves of these three devices at a 5 mV/s scan rate, which can be used to qualitatively reveal their specific capacitance. The larger the specific area of the closed CV pattern, the larger the specific capacitance. For the hybrid supercapacitor device consisting of a mixture of the electrolytes Na_2_SO_4_ and ZnSO_4_, its specific capacitance is larger than of the supercapacitor device with a ZnSO_4_ electrolyte (zinc–ion supercapacitor) or with a Na_2_SO_4_ electrolyte (sodium–ion supercapacitor), which is the smallest. We speculate that this is due to a higher efficiency of divalent Zn^2+^ compared to monovalent Na^+^, while the charge storage mechanism of a device containing a Zn^2+^ electrolyte is based on a Zn^2+^ hybrid supercapacitor. The specific capacitance can be quantified by a CV curve based on equation 1 (Parveen et al., 2016):

(1) $$\begin{array}{l} C=\frac{\int Idt}{mdV} \end{array}$$

where *C, m, V, I, dt* are the specific capacitance (F/g), the mass (g) of the electroactive material, the working voltage window (V), the current (A), and the time differential, respectively. The hybrid supercapacitor device has a specific capacitance of 226 F/g, which is larger than the zinc-ion supercapacitor (209.5 F/g) and higher than the sodium-ion supercapacitor (139.1 F/g).

Figure 3B shows the constant current charge-discharge curves obtained for these three devices at 0.5 A/g current density within the potential arrange of 0–1.6 V. We calculate the specific capacitance according to equation 2 (Meng et al., 2014; Wei et al., 2018c):

(2) $$\begin{array}{l} C=\frac{I\Delta t}{m\Delta V} \end{array}$$

where Δ*V* and Δ*t* are the working potential window (V) and the discharge time (s). In this case, the specific capacitance *C* is directly proportional to the discharge time of Δ*t*. *I/m* is 0.5 A/g and Δ*V* is 1.6 V in Figure 3B, meaning that for the comparison of the specific capacitance, one only needs to assess the size of Δ*t*. The relationship between the specific capacitances can be obtained by a hybrid supercapacitor (190.6 F/g) > zinc–ion supercapacitor (176.8 F/g) > sodium–ion supercapacitor (110.3 F/g). To further characterize the energy storage performance of this hybrid supercapacitor device, we carried out rate performance measurements with different scan rates of CV (Figures 3C,D) and with different current densities of GCD (Figure 3E). When the scan rate increases from 5 to 100 mV/s, the shape of the CV curve shows minor distortion which indicates the good rate performance of the device.

The calculated specific capacitance at different current densities is summarized in Figure 3F. At 0.2 A/g, 212.5 F/g specific capacitance can be achieved for the hybrid supercapacitor, 207.0 F/g for the zinc-ion supercapacitor, and 130.5 F/g for the sodium-ion supercapacitor. When increasing the current density to 20 A/g, 100 F/g specific capacitance is maintained for the hybrid supercapacitor, and 87.5 F/g and 75.0 F/g for the zinc–ion/sodium–ion supercapacitors, respectively. The charge transfer and diffusion impedance of the hybrid supercapacitor device is smaller than that of the zinc/sodium–ion supercapacitor, indicating that the hybrid supercapacitor device has improved electronic and ionic conductivity (Figure S2).

Two key parameters of electrochemical energy storage devices, energy density and power density, can be calculated using the following equations (Chen et al., 2014; Wei et al., 2018a,b):

(3) $$\begin{array}{lll} E & = & \frac{1}{2}CV^{2} \end{array}$$

(4) $$\begin{array}{l} P=\frac{E}{\Delta t} \end{array}$$

where *E* is energy density with a unit of Wh/kg and *P* is power density with a unit of kW/kg, which were calculated and summarized in the Ragone plot of Figure 3G. Our hybrid supercapacitor device shows an *E* of 75.4 Wh/kg at 0.16 kW/kg, which is better than the previously reported zinc-ion supercapacitors and other electrochemical energy storage devices. An energy density of 34.2 Wh/kg is maintained when the power density is increased to 16.0 kW/kg. For the device using ZnSO_4_ as an electrolyte, its energy density is 73.6 Wh/kg at 0.16 kW/kg, and 31.1 Wh/kg at 16.0 kW/kg. In the device using the Na_2_SO_4_ electrolyte, energy density is 46.4 Wh/kg at 0.16 kW/kg, and 26.7 Wh/kg at 16.0 kW/kg. Figure 3H is a comparison chart of the cycle-specific relative capacity of the zinc-ion hybrid supercapacitor with a voltage window from 0 to 1.6 V. The coulombic efficiency of the zinc-ion hybrid supercapacitor reported in this article is close to 100% during the cycling.

The proposed hybrid energy storage device integrates a series of advantages, such as high capacity and energy density, good rate performance, and cycle stability. In addition, the application of non-toxic electrode materials and aqueous electrolytes means that this new system ensures a high degree of safety in terms of human health and the ecological environment. In summary, we propose HMCS electroactive materials with a Zinc-containing electrolyte as suitable for safe, high-rate, and ultra-long-life rechargeable energy storage.

**Figure 4:**
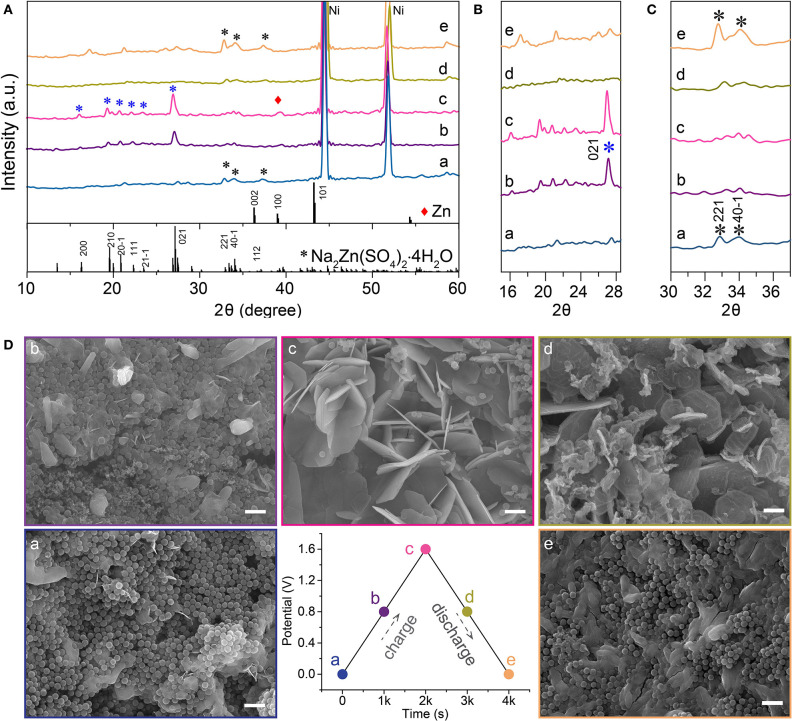
Charge storage mechanism study of zinc-ion hybrid supercapacitors. **(A–C)** XRD spectra of the anode materials at different charge-discharge states. **(D)** SEM images of the anode materials at different charge/discharge states, the scale bars are 1 μm. The star is highligh of the diffraction pattern of Na2Zn(SO4)2 4H2O.

In order to elucidate the charge storage mechanisms of the symmetry zinc-ion hybrid supercapacitor, with HMCS electroactive materials and electrolytes containing ZnSO_4_ and/or Na_2_SO_4_, we performed *quasi in situ* XRD and SEM characterizations of the anode materials at different charge-discharge states, as shown in Figure 4. The charge of the device started at a voltage of 0, followed by being charged to 0.8 V and then 1.6 V. After that, the device was discharged to 0.8 V and finally to 0 V.

**Figure 5:**
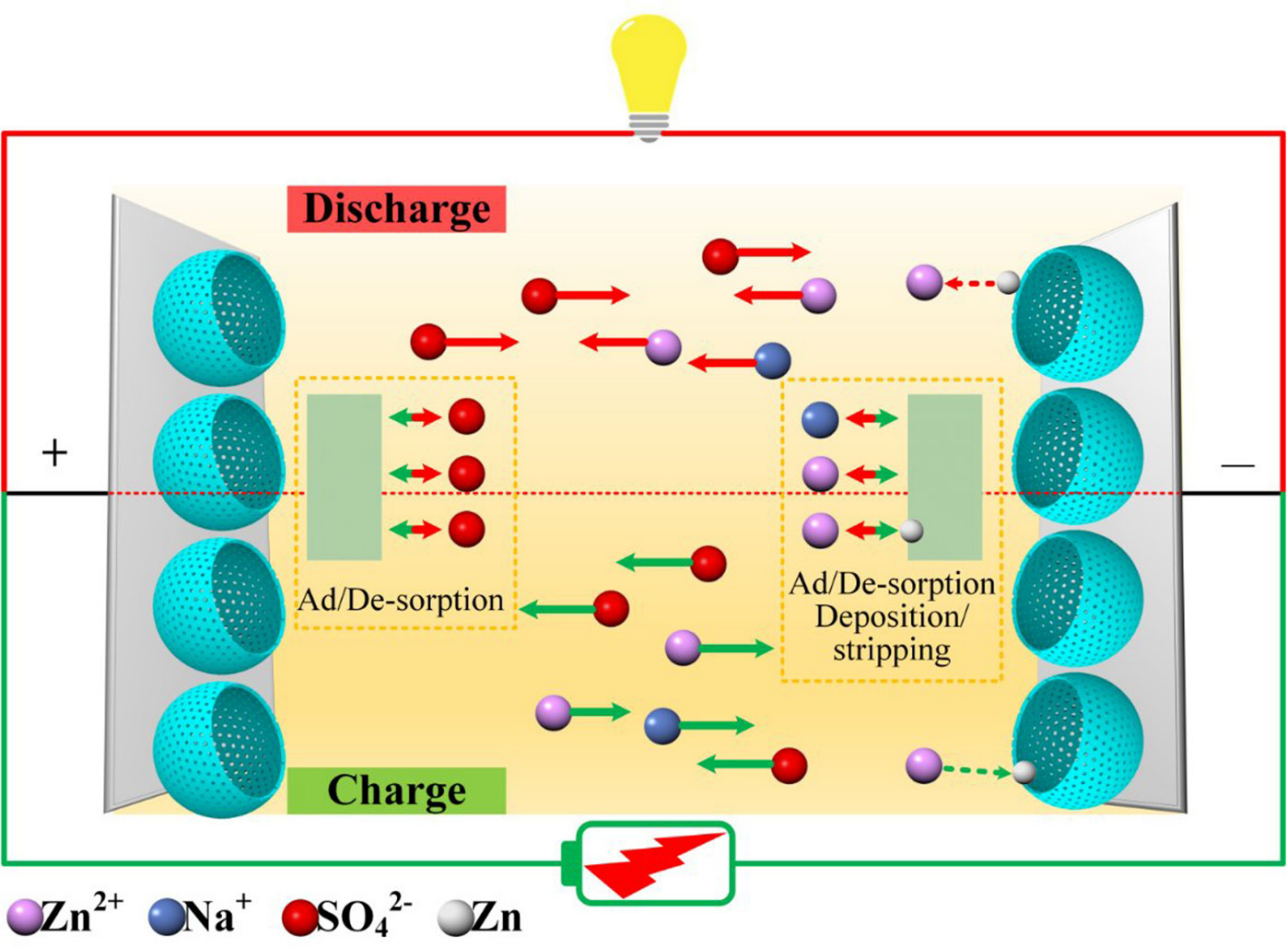
Schematic illustration of the as-proposed device and its working mechanism.

XRD spectra of the device at various states of charge and discharge is shown in Figure 4A. Peaks at 16.3°, 19.6°, 20.9°, 22.3°, 23.5°, 27.1°, 33.4°, 34.1°, and 37.1° for 2θ match well with the (200), (210), $\left(20\bar{1}\right)$, (111), $\left(21\bar{1}\right)$, (021), (221), $\left(40\bar{1}\right)$, and (112) crystal planes of Na_2_Zn(SO_4_)_2_·4H_2_O (PDF No. 51–1557), respectively. The formation of Na_2_Zn(SO_4_)_2_·4H_2_O can be described by equation 5 and its crystallinity is mainly related to the electro–adsorption of Zn^2+^ in the anode. As the charging advances, the diffraction intensities of the (221), $\left(40\bar{1}\right)$, and (112) crystal planes decrease, accompanied by an intensity increase of the (200), (210), $\left(20\bar{1}\right)$, (111), $\left(21\bar{1}\right)$, (021) reflections, where the (021) reflection in particular, indicates a change of the crystallinity of Na_2_Zn(SO_4_)_2_·4H_2_O. In the fully charged state, another diffraction peak at 39.2° can be assigned to the (100) crystal plane of metallic Zn, which demonstrates that a Faradaic reaction following equation 6 takes place at the anode, which is consistent with the occurrence of redox peaks in the CV curve in Figure 3A. As the discharge process proceeds in turn, the (021) peak gradually disappears, accompanied by an intensity recovery of the (221), $\left(40\bar{1}\right)$, and (112) reflections, which can be clearly distinguished from Figures 4B,C. These results indicate that reverse Zn plating/stripping took place on the HMOS anode, as well as Zn^2+^ adsorption/desorption. The SEM images in Figure 4D indicate that the amount of Na_2_Zn(SO_4_)_2_·4H_2_O nanosheets increase along the charging process from A to C, and reversely degrade as the discharge process from C to E. Recharging the device to 1.6 V after *e*, the anode was fully covered with nanosheets like Na_2_Zn(SO_4_)_2_·4H_2_O (Figure S3). The above *quasi in situ* XRD and SEM results prove the excellent cycling stability of Zn-ion hybrid supercapacitors, with its working mechanisms shown in Figure 5 (Dong et al., 2018; Yu et al., 2020).

(5) $$\begin{array}{l} {\text{2Na}}^{+}+{\text{Zn}}^{\text{2}+}+{\text{2SO}}_{\text{4}}^{\text{2}-}+{\text{4H}}_{\text{2}}\text{O}\to {\text{Na}}_{\text{2}}\text{Zn}{\left({\text{SO}}_{\text{4}}\right)}_{\text{2}}\cdot {\text{4H}}_{\text{2}}\text{O} \end{array}$$

(6) $$\begin{array}{l} {\text{Zn}}^{2+}+2{\text{e}}^{-}\to \text{Zn} \end{array}$$

## Conclusions

In conclusion, we propose a novel symmetric zinc-ion hybrid supercapacitor, using hollow mesoporous-carbon nanospheres as electroactive materials and aqueous ZnSO_4_ as an electrolyte. The zinc–ion hybrid supercapacitor shows a high capacity of 212.5 F/g at a current density of 0.2 A/g, a high energy density of 75.4 Wh/kg at a power density of 0.16 kW/kg, a good rate performance (34.2 Wh/kg at high power of 16.0 kW/kg), and remarkable cycling stability (99.4% of the capacity maintained after 2,500 charge/discharge cycles at 2 A/g). This high performance originates from the use of an open carbon structure, which offers a 3-dimensional surface molecular accessibility for reversible ion adsorption/desorption on the cathode and anode, as well as additional Zn/Zn^2+^ deposition/stripping of the anode. We believe that the high performance of zinc-ion hybrid supercapacitor presented here, will be taken forward into the next-generation of energy storage devices.

## Data Availability Statement

The raw data supporting the conclusions of this article will be made available by the authors, without undue reservation.

## Author Contributions

SC, GY, and XZ design and carry out the major experiments and offer original experimental data. SC wrote the manuscript. GY carried out experiments, data analysis, and manuscript revision for peer-reviewing steps. NW, TW, SJ, SQ, TLi, and LD aided the experiments and contribute to the schematic drawing. TLu, JZ, and HaoW perform the analysis with constructive discussions and play an important role in interpreting the results. XC and PA performed TEM characterization and TEM data analysis. XC takes a major part in designing the supercapacitor device configurations and writing the manuscript. HanW supervised the project, contributed to writing the paper, conceived and designed the supercapacitor materials and device, and analyzed all the experiment data. All authors contributed to the article and approved the submitted version.

## Conflict of Interest

The authors declare that the research was conducted in the absence of any commercial or financial relationships that could be construed as a potential conflict of interest.
