# Supplementary material for: Hollow Mesoporous Carbon Spheres for High Performance Symmetrical and Aqueous Zinc-Ion Hybrid Supercapacitor
Source: Front Chem. 2020 Sep 15;8:663. doi: 10.3389/fchem.2020.00663 (PMC7533584; doi:10.3389/fchem.2020.00663)
Supplement: Supplementary file 1 [file Table_1.docx]

**Supporting Information**

**Hollow Mesoporous Carbon Spheres for High Performance Symmetrical and Aqueous Zinc-ion Hybrid Supercapacitor**

Sihan Chen,^a,1^ Gaoqi Yang,^a,1^ Xiaojuan Zhao,^a,1^ Nengze Wang,^a^ Tingting Luo,^b,*^ Xu Chen,^c,*^ Tianci Wu,^a^ Shijie Jiang,^a^ Peter A. van Aken,^c^ Shile Qu,^a^ Tao Li,^a^ Liang Du,^a^ Jun Zhang,^a^ Hanbin Wang,^a,*^ and Hao Wang ^a^

^a^ Hubei Key Laboratory of Ferro & Piezoelectric Materials and Devices, Faculty of Physics and Electronic Science, Hubei University, Wuhan 430062,China

^b^ State Key Laboratory of Advanced Technology for Materials Synthesis and Processing, Wuhan University of Technology, Wuhan 430070, China

^c^ Stuttgart Center for Electron Microscopy, Max Planck Institute for Solid State Research, Stuttgart 70569, Germany

^1^ These authors contributed equally to this work

*Corresponding authors:

Prof. Hanbin Wang, Email: 123272314@qq.com

Dr. Tingting Luo, Email: luotingting27@whut.edu.cn

Dr. Xu Chen, Email: X.Chen@fkf.mpg.de


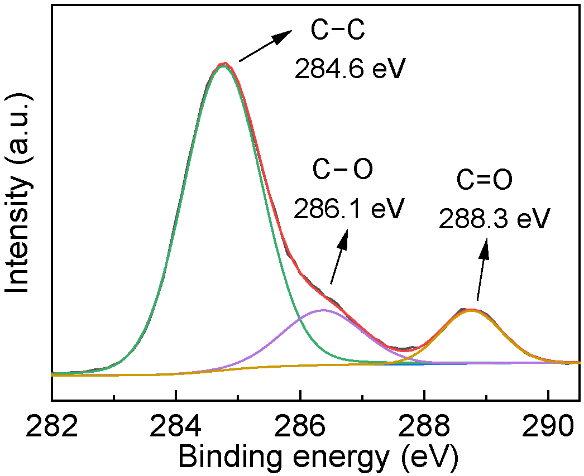


**Figure S1** XPS spectrum of the as–synthesized hollow mesoporous carbon spheres.

**Figure S2** Nyquist plots of the devices within electrolytes of ZnSO_4_, Na_2_SO_4_ and ZnSO_4_/Na_2_SO_4_ mixture, respectively.


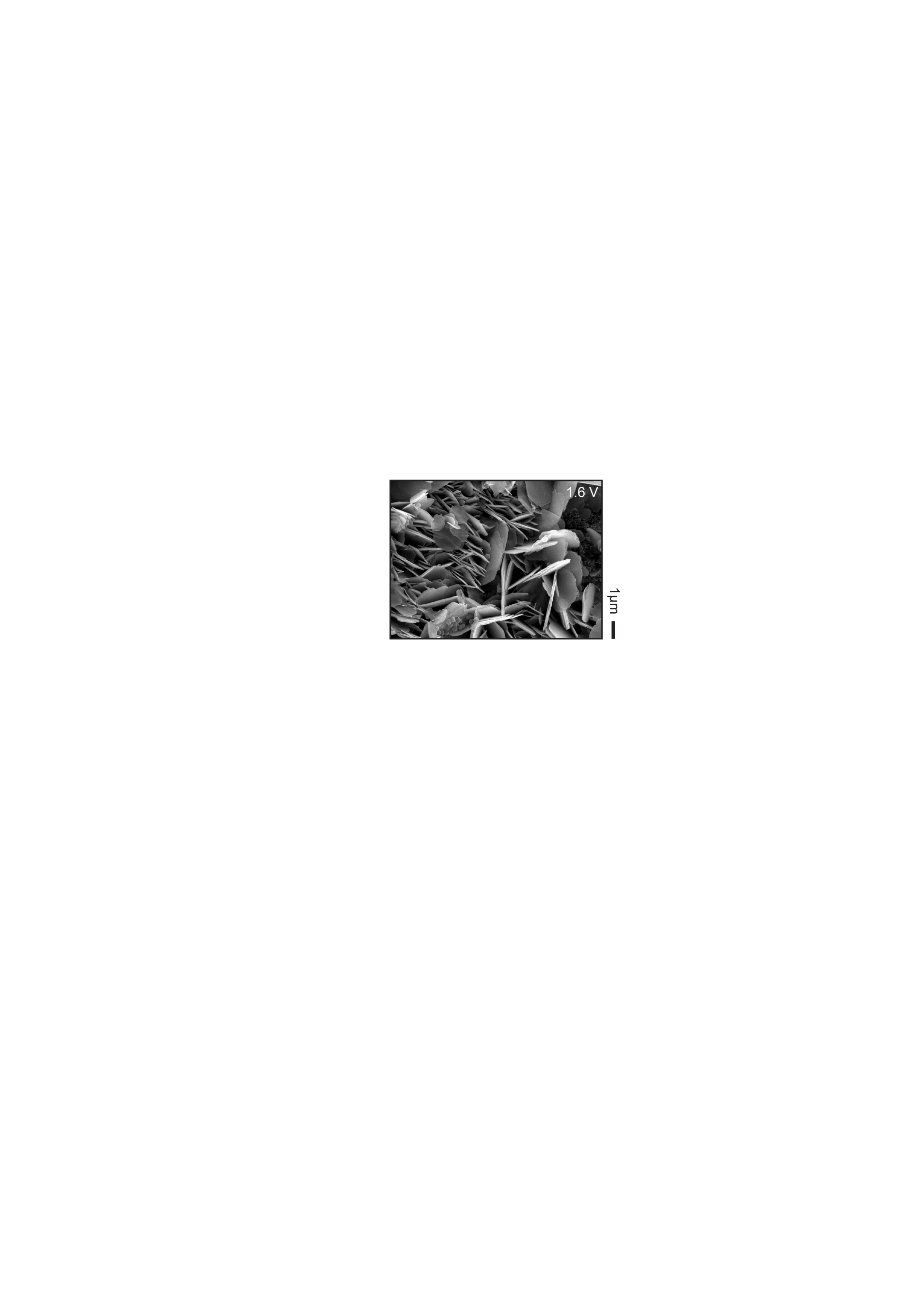


**Figure S3** SEM images of the anode materials when recharged into 1.6 V, follow the charge/discharge process of Fig. 4D of the manuscript.
